# Protein and transcriptional biomarker profiling may inform treatment strategies in lower respiratory tract infections by indicating bacterial–viral differentiation

**DOI:** 10.1128/spectrum.02831-23

**Published:** 2024-09-13

**Authors:** Dhanasekaran Sivakumaran, Synne Jenum, Dagfinn Lunde Markussen, Sondre Serigstad, Aashish Srivastava, Christina Skår Saghaug, Elling Ulvestad, Siri Tandberg Knoop, Harleen M. S. Grewal

**Affiliations:** 1 Department of Clinical Science, Bergen Integrated Diagnostic Stewardship Cluster, University of Bergen, Bergen, Norway; 2 Department of Infectious Diseases, Oslo University Hospital, Oslo, Norway; 3 Emergency Care Clinic, Haukeland University Hospital, Bergen, Norway; 4 Department of Microbiology, Haukeland University Hospital, Bergen, Norway; 5 Department of Clinical Medicine, University of Bergen, Bergen, Norway; 6 Genome Core-Facility, Clinical Laboratory (K2), Haukeland University Hospital, University of Bergen, Bergen, Norway; Uniwersytet Medyczny w Bialymstoku, Bialystok, Poland

**Keywords:** acute respiratory infections, bacterial vs viral diagnosis, host-based biomarkers, biosignatures, antibiotic treatment

## Abstract

Lower respiratory tract infections (LRTIs) remain a significant global cause of infectious disease-related mortality. Accurate discrimination between acute bacterial and viral LRTIs is crucial for optimal patient care, prevention of unnecessary antibiotic prescriptions, and resource allocation. Plasma samples from LRTI patients with bacterial (*n* = 36), viral (*n* = 27; excluding SARS-CoV-2), SARS-CoV-2 (*n* = 22), and mixed bacterial–viral (*n* = 38) etiology were analyzed for protein profiling. Whole-blood RNA samples from a subset of patients (bacterial, *n* = 8; viral, *n* = 8; and SARS-CoV-2, *n* = 8) were analyzed for transcriptional profiling. Lasso regression modeling identified a seven-protein signature (CRP, IL4, IL9, IP10, MIP1α, MIP1β, and TNFα) that discriminated between patients with bacterial (*n* = 36) vs viral (*n* = 27) infections with an area under the curve (AUC) of 0.98. When comparing patients with bacterial and mixed bacterial–viral infections (antibiotics clinically justified; *n* = 74) vs patients with viral and SARS-CoV-2 infections (antibiotics clinically not justified; *n* = 49), a 10-protein signature (CRP, bFGF, eotaxin, IFNγ, IL1β, IL7, IP10, MIP1α, MIP1β, and TNFα) with an AUC of 0.94 was identified. The transcriptional profiling analysis identified 232 differentially expressed genes distinguishing bacterial (*n* = 8) from viral and SARS-CoV-2 (*n* = 16) etiology. Protein–protein interaction enrichment analysis identified 20 genes that could be useful in the differentiation between bacterial and viral infections. Finally, we examined the performance of selected published gene signatures for bacterial–viral differentiation in our gene set, yielding promising results. Further validation of both protein and gene signatures in diverse clinical settings is warranted to establish their potential to guide the treatment of acute LRTIs.

**IMPORTANCE:**

Accurate differentiation between bacterial and viral lower respiratory tract infections (LRTIs) is vital for effective patient care and resource allocation. This study investigated specific protein signatures and gene expression patterns in plasma and blood samples from LRTI patients that distinguished bacterial and viral infections. The identified signatures can inform the design of point-of-care tests that can aid healthcare providers in making informed decisions about antibiotic prescriptions in order to reduce unnecessary use, thereby contributing to reduced side effects and antibiotic resistance. Furthermore, the potential for faster and more accurate diagnoses for improved patient management in acute LRTIs is compelling.

## INTRODUCTION

Lower respiratory tract infections (LRTIs) are a leading cause of mortality worldwide, with rates increasing with aging populations in many developed countries (1, 2). Global efforts to reduce the burden of LRTIs depend on information about associated pathogens (1), as timely and appropriate use of antibiotic therapy is essential to improve outcomes, reduce hospital admissions, and contribute to antibiotic stewardship by minimizing un-targeted broad-spectrum antibiotics (3).

Early detection of respiratory pathogens has been linked to shorter hospital stays, improved antimicrobial stewardship, and better cohort assignation for preventing nosocomial infections and outbreaks (4–8). Recent developments in molecular diagnostic methods, like nucleic acid amplification tests (NAATs), have improved the ability to identify viruses in respiratory samples, suggesting that viruses are causative agents in a significant proportion of adult community-acquired pneumonia (CAP) cases (9). Nevertheless, 30%–64% of LRTI patients do not get an appropriate microbial diagnosis (10–19). This may lead to antibiotic overuse in the case of viral etiology and improper use when empirical treatments are too broad or inactive against the causative bacteria.

Molecular diagnostics applied to assess host immune responses (20) have emerged as a promising supplement to pathogen-based diagnostics in discriminating viral from bacterial LRTIs (21, 22). Such diagnostics might supplement traditional measurements of C-reactive protein (CRP), leucocyte counts, and procalcitonin (PCT) in decisions of antibiotic initiation and/or withdrawal (23, 24) and may also provide guidance to adjunctive host-directed therapies as demonstrated in patients with COVID-19 (25). After the emergence of SARS-CoV-2, several studies have tried to distinguish between the host response in COVID-19 and the host response in RTIs of other microbial causes (24, 26–28). This is important to direct the development of specific anti-inflammatory or antiviral treatment strategies. From an antibiotic stewardship perspective, however, it may also be useful to exploit similarities in the host response to SARS-CoV-2 and other viral etiologies in order to identify a common viral comparator to bacterial infections.

The present study investigates host biomarker signature(s) in adult LRTI patients with the aim of distinguishing patients in need of antibiotic therapy. We performed protein profiling to discriminate patients with viral etiology (including SARS-CoV-2) from patients with bacterial etiology (including patients with bacterial and viral coinfections). We also compared the host transcriptional profile of patients with LRTI caused by SARS-CoV-2, other viral etiologies, and bacterial etiology, in order to identify differentially expressed genes including those involved in the activation of host immune pathways. Finally, we evaluated promising published gene signatures (29–32) that effectively discriminate between bacterial and viral etiology in our transcriptional data set.

## RESULTS

### Baseline characteristics of study participants

**Fig 1:**
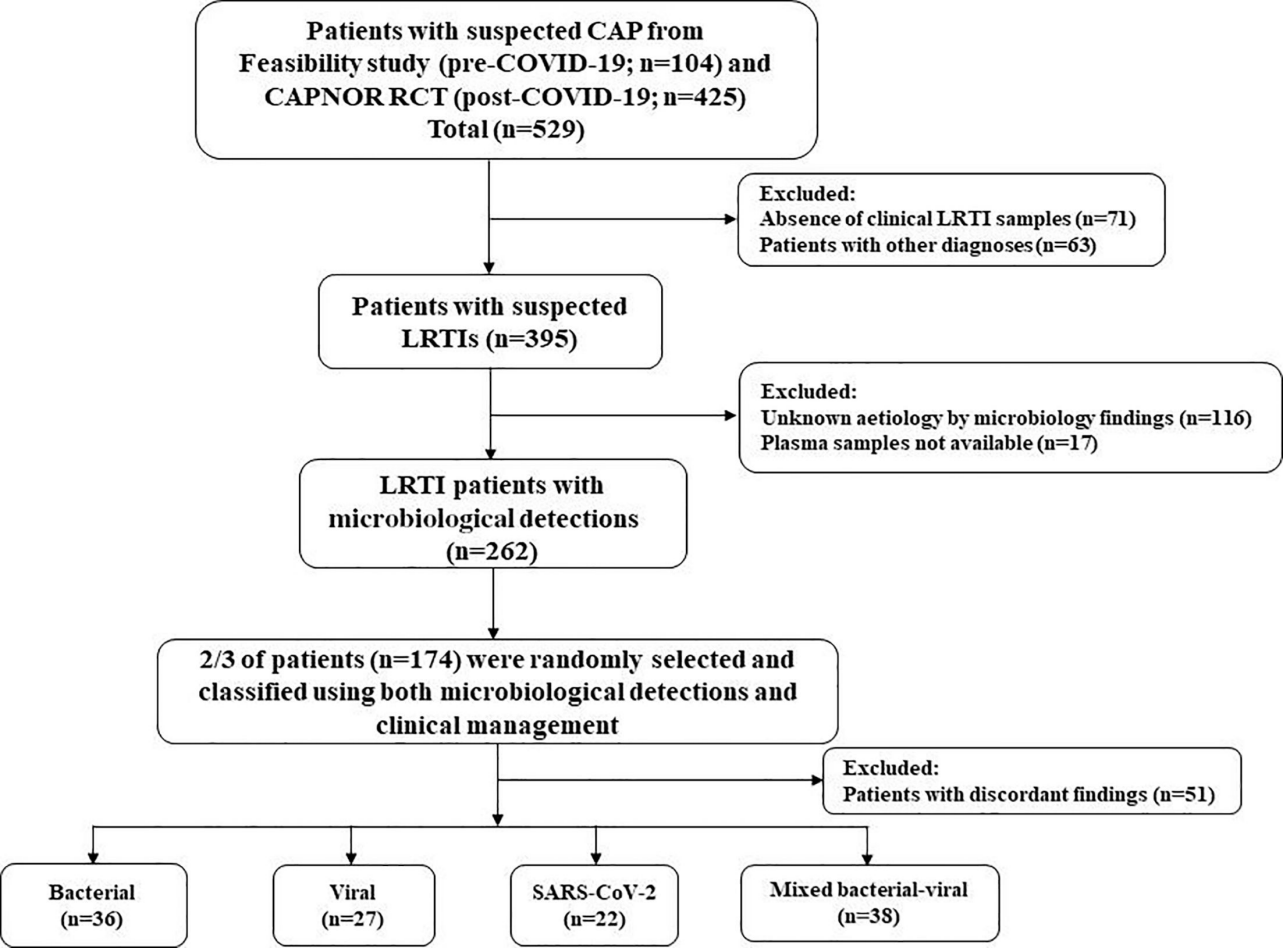
Study flowchart. ^*^Discordant findings defined as (1) detection of only viral (including SARS-CoV-2) etiology, but antibiotics administered for ≥120 hours in the hospital, or (2) detection of bacteria and/or mixed bacterial–viral etiology, but antibiotics administered for <120 hours (including post-discharge).

A total of 123 LRTI patients were selected and included (Fig. 1). Patients with SARS-CoV-2 had a comparatively younger median age of 58.5 years and a lower proportion of females than patients with other etiologies. Furthermore, 68.2% of the patients with SARS-CoV-2 etiology received no or negligible amounts of antibiotics (Table 1).

**TABLE 1:** Baseline characteristics of study participants^*a*^

	Bacterial etiology (*n* = 36)	Viral etiology (*n* = 27)	SARS-CoV-2 (*n* = 22)	Mixed bacterial–viral etiology (*n* = 38)
Age in years (median with IQR)	73 (60.5–78)	66 (52–74.5)	58.5 (47–76.8)	68 (52.5–80.0)
Female (%)	17 (47.2)	16 (59.3)	6 (27.3)	25 (65.8)
Patients diagnosed with LRTIs				
Confirmed CAP^*b*^ (%)	36 (100.0)	5 (18.5)	15 (68.2)	36 (94.7)
Other respiratory tract infections (%)	0 (0.0)	15 (55.5)	6 (27.3)	2 (5.2)
Infectious exacerbation of COPD (%)	0 (0.0)	7 (25.9)	1 (4.5)	0 (0.0)
Laboratory findings				
C-reactive protein (mg/L; median with IQR)	141 (69–258.3)	38 (22.5–57.5)	62.5 (27.8–131.5)	180.0 (108–245)
Procalcitonin (µg/L; median with IQR)	0.2 (0.8–0.44)	0.01 (0.0–0.23)	0.19 (0.0–0.46)	0.28 (0.15–0.74)
Antibiotic usage				
Antibiotics not given or if given ≤8 hours (%)	0 (0.0)	10 (37.0)	15 (68.2)	0 (0.0)

^*a*^ IQR, interquartile range; COPD, chronic obstructive pulmonary disease.

^*b*^ Clinically confirmed CAP, of which 75 out of 92 were radiologically confirmed cases.

### Host biomarker profiling

#### Protein profiling

**Fig 2:**
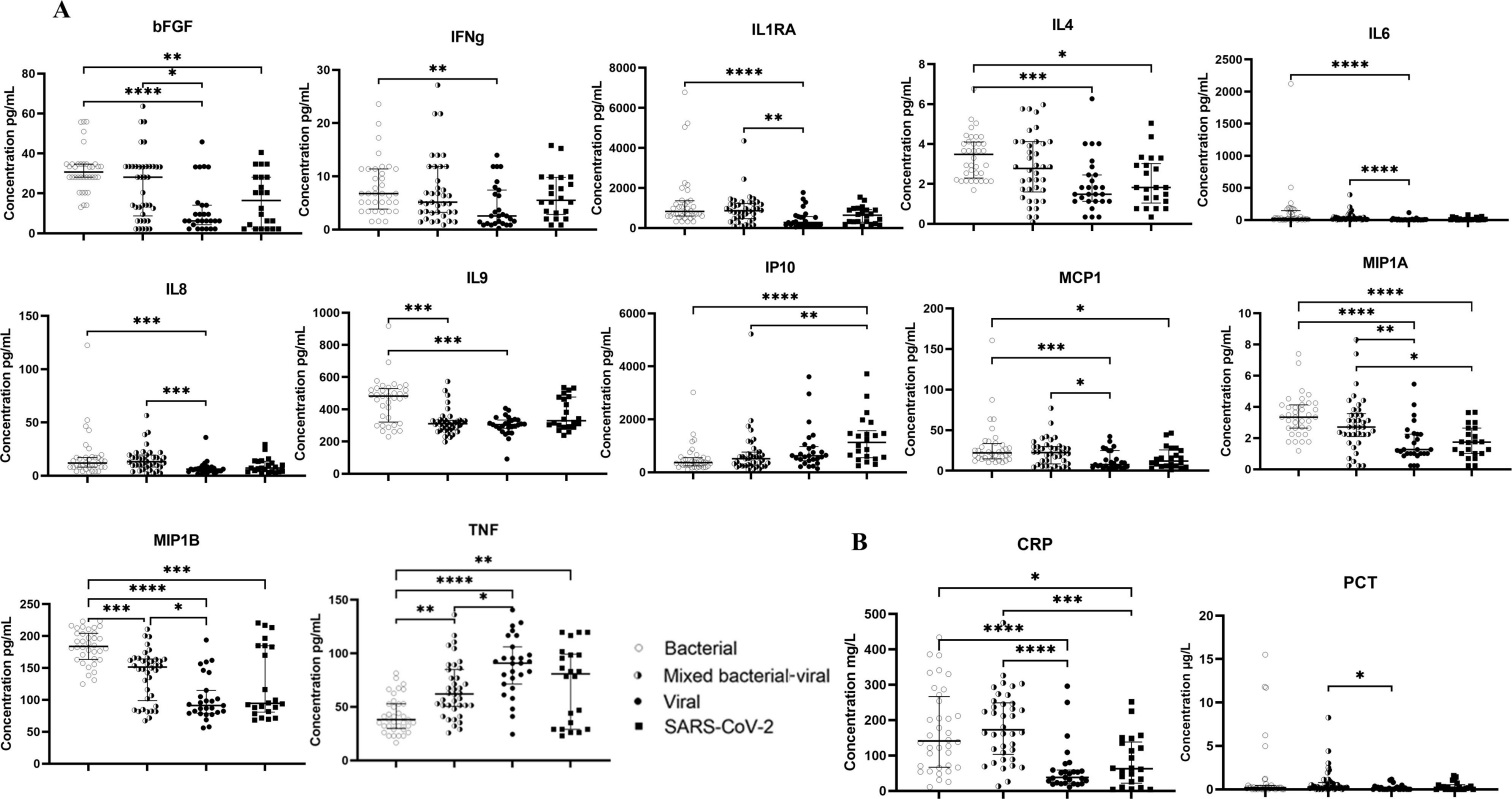
(**A**) Scatter plot graph depicting median cytokine/chemokine concentrations (pg mL^−1^) in plasma samples from LRTI patients with bacterial, mixed bacterial–viral, viral, and SARS-CoV-2 etiologies. (**B**) Scatter plot graph depicting median CRP (mg L^−1^) and procalcitonin (µg L^−1^) concentrations from the serum samples. The Kruskal–Wallis test with Dunn’s *post hoc* correction was applied. *P*-values <0.05 (*), <0.01 (**), <0.001 (***), and <0.0001 (****) were considered to be significant.

Six of the 27 analytes [interleukin (IL)2, IL5, IL15, IL17, granulocyte-macrophage colony-stimulating factor (GM-CSF), and vascular endothelial growth factor (VEGF)] were detectable in <40% of the samples and, therefore, excluded from further analyses. Pairwise comparisons of protein levels between patients with bacterial, viral (excluding SARS-CoV-2), SARS-CoV-2, and mixed bacterial–viral etiology were performed and showed higher concentrations of macrophage inflammatory protein (MIP)1β and lower concentrations of tumor necrosis factor (TNF)α in patients with bacterial etiology compared to all other groups. Concentrations of basic fibroblast growth factor (bFGF), IL4, monocyte chemotactic protein (MCP-1), and MIP1α were higher in patients with bacterial etiology compared to patients with viral and SARS-CoV-2 etiology. Furthermore, patients with SARS-CoV-2 had higher concentrations of IP10 compared to patients with bacterial and mixed bacterial–viral etiologies. Patients with viral etiology had lower concentrations of IL1 receptor antagonist (IL1RA), IL6, and IL8 compared to patients with bacterial and mixed bacterial–viral etiologies and lower concentrations of interferon (IFN)γ and IL9 compared to patients with bacterial etiology (Fig. 2A). Also, patients with either viral or SARS-CoV-2 etiology had reduced concentrations of CRP compared to patients with bacterial and mixed bacterial–viral etiology, whereas patients with viral etiology had reduced concentrations of PCT compared to patients with mixed bacterial–viral etiology (Fig. 2B).

**Fig 3:**
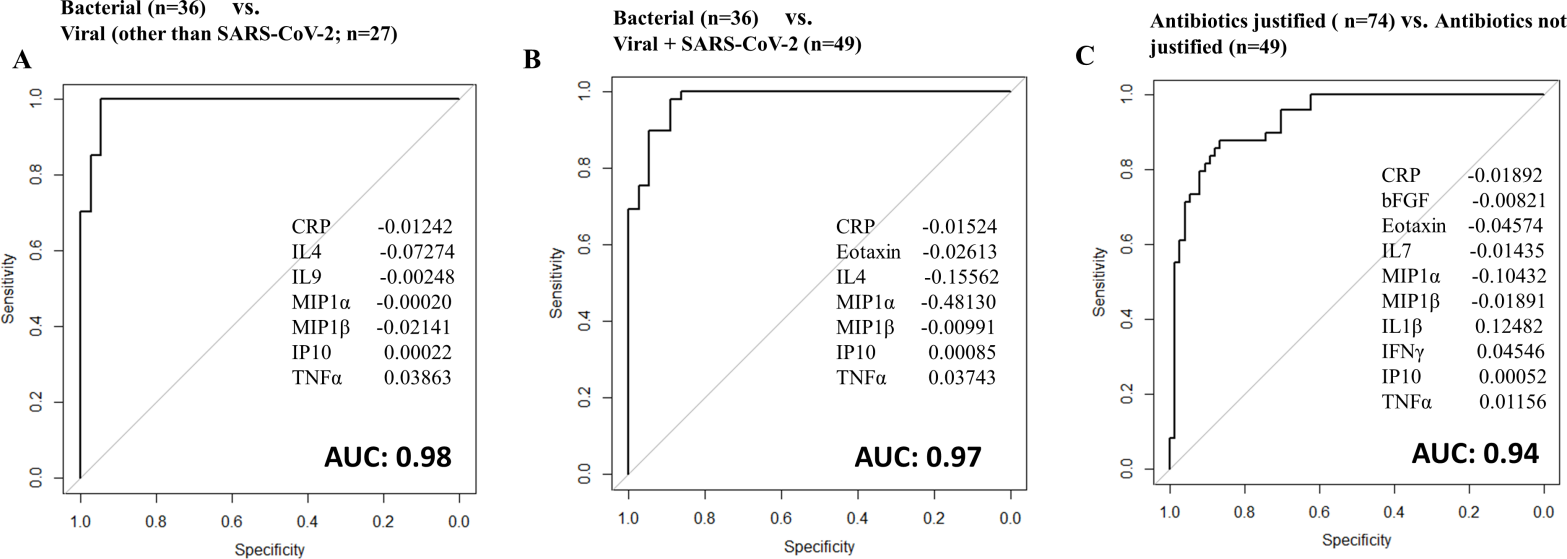
Receiver operator characteristic (ROC) curves for protein signatures that distinguish (**A**) patients with bacterial from viral etiology, (**B**) patients with bacterial from viral and SARS-CoV-2 etiology, and (**C**) patients with bacterial and mixed bacterial–viral etiology (“antibiotics justified”) from patients with viral and SARS-CoV-2 etiology (“antibiotics not justified”).

Aiming to identify the best discriminatory signature between patients classified with bacterial versus viral etiology, we applied the Lasso regression model. First, we compared patients with viral (excluding SARS-CoV-2) to bacterial etiology and identified a seven-protein signature (CRP, IL4, IL9, IP10, MIP1α, MIP1β, and TNFα) with an area under the curve (AUC) of 0.98 (95%CI: 0.97–1.00), a sensitivity of 94.4% (95%CI: 89.3%–99.3%), and a specificity of 92.6% (95%CI: 75.7%–99.1%; Fig. 3A). Then, we did the same analysis with the SARS-CoV-2 patients replacing other viral etiologies (SARS-CoV-2 compared to bacterial etiology) and identified a seven-protein signature (CRP, eotaxin, IL4, IL7, IP10, MIP1a, and TNFα) with an AUC of 0.97 (95%CI: 0.94–1.00), a sensitivity of 91.7% (95%CI: 77.5%–98.3%), and a specificity of 81.8% (95%CI: 60.0%–94.8%; Fig. S1A).

Next, the cases with viral or SARS-CoV-2 etiology were merged into one group and compared to bacterial etiology. The Lasso regression model found a seven-protein signature (CRP, eotaxin, IL4, IP10, MIP1α, MIP1β, and TNFα) with an AUC of 0.97 (95%CI: 0.95–1.00), a sensitivity of 88.9% (95%CI: 73.9%–96.9%), and a specificity of 89.8% (95%CI: 77.8%–96.6%; Fig. 3B). A seven-protein signature with IL9 replacing eotaxin had equal performance (CRP, IL9, IL4, IP10, MIP1α, MIP1β, and TNFα) with an AUC of 0.97 (95%CI: 0.96–1), a sensitivity of 88.9% (95%CI: 73.9%–96.9%), and a specificity of 89.8% (95%CI: 77.8%–96.6%; Fig. S1B).

Furthermore, we aimed to identify discriminatory signatures between the more clinically relevant groups, namely, those requiring antibiotics versus those who do not. Thus, patients with bacterial etiology and those with mixed bacterial–viral etiology were merged into one group named “antibiotics justified” (*n* = 74), whereas patients with viral etiology including those with SARS-CoV-2 were merged into one group named “antibiotics not justified” (*n* = 49). The best discrimination was obtained by a 10-protein signature identified by the Lasso regression model (CRP, bFGF, eotaxin, IFNγ, IL1β, IL7, IP10, MIP1α, MIP1β, and TNFα) with an AUC of 0.94 (95%CI: 0.90–0.98), a sensitivity of 91.9% (95%CI: 83.2%–97.0%), and a specificity of 83.7% (95%CI: 70.3%–92.7%; Fig. 3C).

Last, we evaluated the performance of the identified seven-protein signature distinguishing bacterial (*n* = 36) from viral and SARS-CoV-2 (*n* = 20) infections specifically in patients with CAP. The resulting AUC was 0.96 (95%CI: 0.94–1), with a sensitivity of 91.7% (95%CI: 77.5%–98.3%) and a specificity of 80.0% (95%CI: 56.3%–94.3%; Fig. S2A). Additionally, a comparable evaluation in CAP patients was carried out for the 10-protein signature that distinguished between “antibiotics justified” (bacterial and mixed bacterial–viral infections; *n* = 72) vs “antibiotics not justified” (viral and SARS-CoV-2 infections; *n* = 20). The resulting AUC was 0.90 (95%CI: 0.83–0.96), with a sensitivity of 91.7% (95%CI: 82.7%–96.9%) and a specificity of 65.0% (95%CI: 40.8%–84.6%) (Fig. S2B). Many misclassified CAP infections were SARS-CoV-2 cases (4 out of a total of 7 misclassifications in the 7-protein signature evaluation and 7 out of 13 misclassifications in the 10-protein signature evaluation).

#### Transcriptional profiling

**Fig 4:**
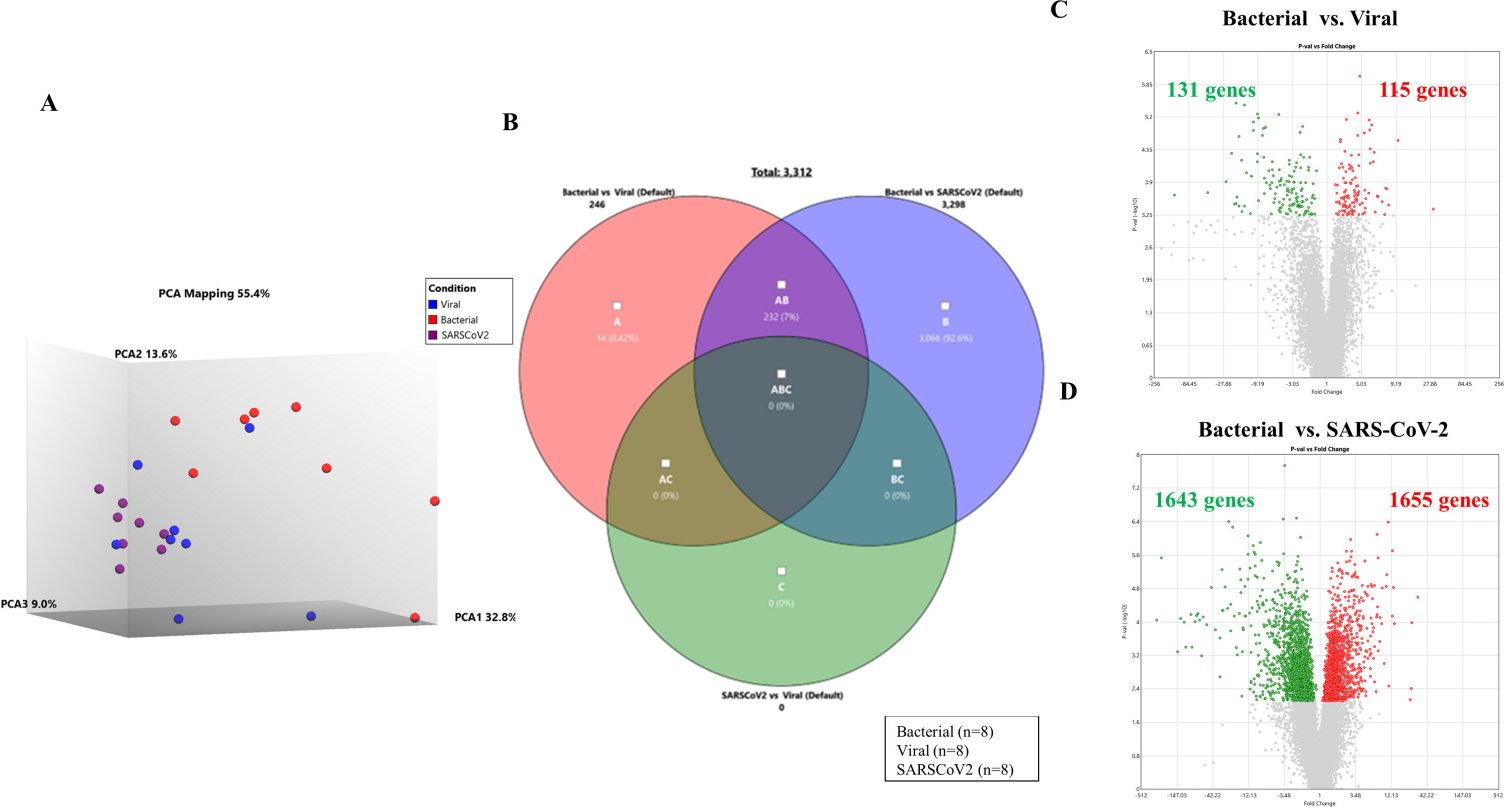
(**A**) PCA mapping of all expressed genes in the whole blood from LRTI patients with bacterial, viral, and SARS-CoV-2 etiologies. (**B**) The Venn diagram shows the distribution of differentially expressed genes (DEGs) identified between three comparisons [bacterial vs viral (pink circle), bacterial vs SARS-CoV-2 (purple circle), and viral vs SARS-CoV-2 (green circle)]. (**C and D**) Volcano plot comparing LRTI patients with (**C**) bacterial (*n* = 8) vs viral (*n* = 8) etiology and (D) bacterial (*n* = 8) vs. SARS-CoV-2 (*n* = 8) etiology. The fold change indicates the mean expression level for each gene. Each dot represents one gene. Gray dots represent no significant DEGs between patients with bacterial etiology and patients with viral etiology; the green dots represent down-regulated genes, and the red dots represent up-regulated genes.

Twenty-four patients were selected for transcriptomic profiling. Table 2 shows the overview of microbial detections in their lower respiratory tract (LRT) samples. Unsupervised principal component analysis (PCA) of differentially expressed genes was performed with patient groups separated based on etiology: bacterial (*n* = 8) vs viral (*n* = 8) vs SARS-CoV-2 (*n* = 8), showing a majority of patients with viral and SARS-CoV-2 etiology clustered in one group away from patients with bacterial etiology (Fig. 4A). When comparing bacterial vs viral etiology, 246 genes (*P* < 0.05) were differentially expressed (Fig. 4B and C), whereas 3,298 genes (*P* < 0.05) were differentially expressed between bacterial and SARS-CoV-2 etiology (Fig. 4B and D). No genes were differentially expressed when comparing the viral vs SARS-CoV-2 groups (Fig. 4B). Notably, 232 genes were represented in both comparisons (i.e., bacterial vs viral and bacterial vs SARS-CoV-2) (Table S1). Of these genes, 126 were down-regulated and 106 were up-regulated in patients with bacterial etiology compared to patients with viral and SARS-CoV-2 etiology.

**TABLE 2:** Microbial detections in samples used for transcriptomic profiling

Group	Microbial detection(s)				
Bacterial	*Haemophilus influenzae*	*Mycoplasma pneumoniae*	*H. influenzae/Streptococcus pneumoniae*	*Staphylococcus aureus/Escherichia coli/Klebsiella oxytoca*	*H. influenzae/Moraxella catarrhalis/E. coli*
Patients (*n*=)	3	2	1	1	1
Viral	Influenza A virus	Human metapneumovirus	Influenza A virus/respiratory syncytial virus		Coronavirus other than SARS-CoV-2
Patients (*n*=)	3	3	1		1
COVID-19	SARS-CoV-2				
Patients (*n*=)	8				

**Fig 5:**
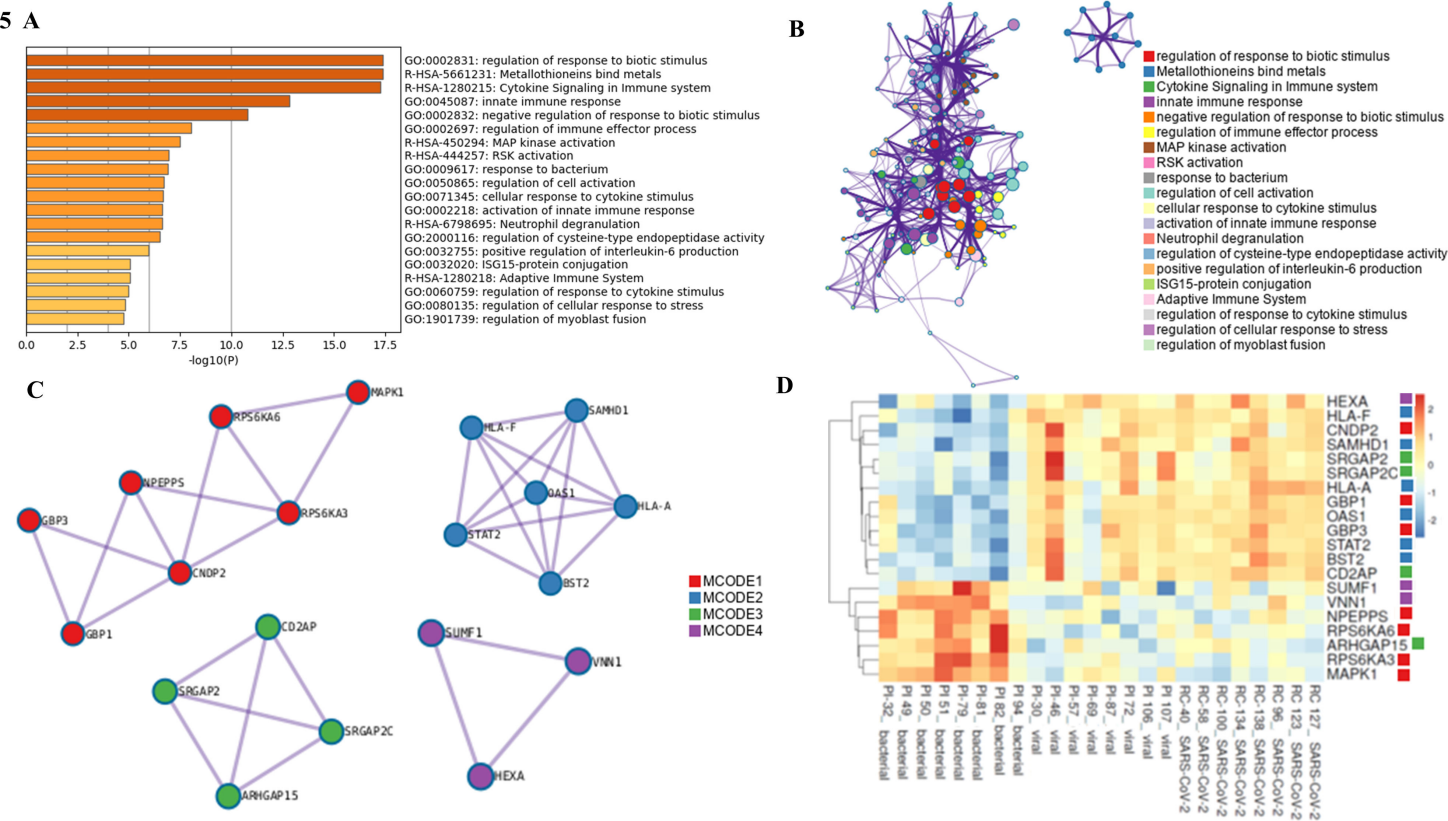
(**A**) Bar graph of the top 20 enriched terms among the 232 genes represented in comparison of both bacterial vs viral and bacterial vs SARS-CoV-2 etiologies, colored by *P*-values. (**B**) Network plot of the top 20 enriched terms visualized using Cytoscape (http://www.cytoscape.org). Each term is represented by a circle node, where its size is proportional to the number of input genes falling under that term, and its color represents its cluster identity. (**C**) Protein–protein interaction networks identified using the MCODE algorithm. Enrichment analysis was applied to each MCODE network to explore their biological relationships, where the top 3 best *P*-value terms (if any) are presented: **MCODE1** (consists of seven genes): (1) R-HSA-444257—RSK activation (*P* < 0.00001); (2) R-HSA-442742—CREB1 phosphorylation through NMDA receptor-mediated activation of RAS signaling (*P* < 0.00001); (3) R-HSA-437239—recycling pathway of L1 (*P* < 0.00001); **MCODE2** (consists of six genes): (1) R-HSA-909733—interferon alpha/beta signaling (*P* < 0.00001); (2) R-HSA-913531—interferon signaling (*P* < 0.00001); (3) GO:0045824—negative regulation of innate immune response (*P* < 0.00001); **MCODE3** (consists of four genes): (1) GO:0051056—regulation of small GTPase-mediated signal transduction (*P* < 0.00001); (2) GO:0050808—synapse organization (*P* < 0.00001); (3) GO:0120031—plasma membrane-bounded cell projection assembly (*P* = 0.00001); **MCODE4** (consists of three genes): no enriched terms reported. (**D**) Heatmap generated for the 20 differentially expressed genes identified across all four MCODE networks, with columns representing samples and rows representing genes.

Analysis of the set of 232 differentially expressed genes using the Metascape tool provided further insight into the biological processes involved (Fig. 5). Figure 5A shows a bar graph of the top 20 highly enriched Gene Ontology (GO) terms across the input gene list ranked by their *P*-values, and Fig. 5B visualizes a subset of the most representative terms converted into a network layout where nodes of the same color belong to the same cluster. Then, to compile a set of relevant genes for direct comparison among the patients with bacterial, viral, and SARS-CoV-2 etiology, Metascape’s Molecular Complex Detection (MCODE) algorithm was used to extract protein–protein interaction networks (Fig. 5C). Four MCODE networks with a total of 20 differentially expressed genes were identified. Finally, these 20 genes were selected for hierarchical clustering and heatmap analysis (Fig. 5D).

#### Validation of the Xu-2, Li-3, Rao-8, and Ravichandran-10 signatures

Due to the limited sample size, we did not perform *de novo* regression analyses to identify possible discriminatory signatures in our transcriptional data set. Instead, we explored the performance of promising previously published gene signatures (Table 3). The Rao-8 and Ravichandran-10 gene signatures yielded excellent discriminatory capabilities in distinguishing bacterial (*n* = 8) from viral and SARS-CoV-2 (*n* = 16) etiologies with AUCs of 0.99 (0.99–1.00) and corresponding sensitivity and specificity of 100.0%, whereas the Xu-2 and Li-3 signatures demonstrated less robust discriminatory power as depicted in Table 3.

**TABLE 3:** Validation of the four selected gene signatures: discriminating patients with bacterial (*n* = 8) from viral and SARS-CoV-2 (*n* = 16) etiologies

Study	Sensitivity in % (95%CI)	Specificity in % (95%CI)	AUC (95%CI)	Accuracy in %
Xu et al*.*				
**2-gene signature** *IFI44L, PI3*	75.0 (34.9–96.8)	93.8 (69.8–99.8)	0.91 (0.78–0.1.00)	87.5
Li et al*.*				
**3-gene signature** *HERC6, IGF1R, NAGK*	87.5 (47.4–99.7)	93.8 (69.8–99.8)	0.97 (0.91–0.1.00)	91.7
Rao et al*.*				
**8-gene signature** *EPI3, FCER1A, HESX1, ICAM1, IFI27, JUP, SMARCD3, SUCLG2*	100.0 (63.1–100.0)	100.0 (79.4–100.0)	0.99 (0.99–1.00)	100.0
Ravichandran et al*.*				
**10-gene signature** *DNMT1, EPSTI1, GYG1, HK3, IFI27, IFI44, ISG15, MMP9, MX1, PRF1*	100.0 (63.1–100.0)	100.0 (79.4–100.0)	0.99 (0.99–1.00)	100.0

## DISCUSSION

While a wide variety of viral and bacterial pathogens can cause LRTIs (33), it is often not possible to distinguish with certainty between bacterial and viral pathogenesis based on signs and symptoms alone. Even though modern NAATs like PCR possess excellent sensitivity for the identification of respiratory viruses from naso- and oropharyngeal swabs, a bacterial coinfection may still be present in the LRT. The precision of microbiological diagnostics is hampered by the difficulty in obtaining representative and good-quality LRT samples, especially from severely ill patients, which likely leads to inappropriate antibiotic use due to the fear of missing a bacterial diagnosis (34). Furthermore, most of the bacteria that may be involved in LRTIs are also potential colonizers of the upper airways, and bacterial detection is, therefore, not necessarily clinically relevant.

In this study, we have utilized biobanked plasma samples to examine selected protein biomarkers, with the aim to identify protein signatures that may contribute to the precise discrimination between patients with a viral or bacterial LRTI, as an attempt to contribute to the optimization of treatment strategies. We identified protein signatures that could differentiate patients requiring antibiotics (i.e., bacterial and mixed bacterial–viral etiology) from patients not requiring antibiotics (i.e., viral + SARS-CoV-2 etiology) with promising performances. Notably, five proteins (CRP, IP10, MIPα, MIP1β, and TNFα) were represented in the identified 7- and 10-protein signatures.

Commercial protein biosignature tests for bacterial–viral differentiation are available (35), but their diagnostic accuracy across a broad range of pathogens is not yet determined (36). The FebriDx test targets CRP and Myxovirus resistance protein A, where the latter is a marker of interferon-induced antiviral host response, and has been found to have a high accuracy in terms of indicating the presence of a viral infection in hospitalized adults with SARS-CoV-2 (37). The lack of a specific bacterial marker does, however, imply that its approach can fall through in the case of a bacterial coinfection. Thus, in a hospital setting with NAAT capacity for a broad range of viral pathogens, its use may be less relevant. The MeMed test includes three markers, whereof two are viral-induced markers: interferon-gamma inducible protein-10 (IP-10; also known as CXCL10) and tumor necrosis factor-related apoptosis-inducing ligand (TRAIL), in addition to CRP. However, while TRAIL levels are reduced in bacterial infections, this may also be the case in some severe viral infections, again implying a shortcoming in terms of ruling out a bacterial coinfection. Interestingly, the MeMed combination score has recently also been validated as a tool to predict outcomes in COVID-19 patients, exploiting this phenomenon (38). There are, to our knowledge, no reports on the performance of the MeMed test in cohorts that have included SARS-CoV-2 patients.

In this study, SARS-CoV-2 patients were included, and both were investigated separately and as a merged category with other viral etiologies to explore possible different hallmarks in the host response comparisons to bacterial LRTIs. Of note, the protein signature identified without SARS-CoV-2 patients provided excellent discriminatory power in accurately classifying bacterial and viral etiologies, while the signature identified with SARS-CoV-2 patients included was found to be somewhat inferior. This may be due to a lack of control for confounding variables, e.g., different disease severities, but may also be due to limitations in the analytes available for protein profiling, which were pre-defined. To account for disease severity, we evaluated our protein signatures’ ability to distinguish between bacterial and viral infections among our CAP cases. The results showed similar effective performance in terms of the AUC when comparing the combined viral groups (other viruses + SARS-CoV-2) against the bacterial group, both individually and combined with mixed bacterial–viral infections (“antibiotics justified”). However, the specificity of the signatures, which relates to accurately classifying a viral infection, was lower. Notably, many misclassifications were SARS-CoV-2 patients. Regrettably, due to the small number of CAP cases with other viral infections, we were unable to investigate this further.

To investigate host response in the different microbiological categories in more depth, we performed transcriptomic profiling on a selected subcohort. Surprisingly, no genes were differentially expressed when comparing the viral vs SARS-CoV-2 groups by transcriptional profiling. Although we used the Metascape tool to select 20 differentially expressed genes for heatmap analysis, we acknowledge that the transcriptomic analysis was conducted in a limited sample size. Thus, we chose to evaluate previously published gene signatures that were identified from larger data sets. The assessment of the previously published gene signatures (Xu-2, Li-3, Rao-8, and Ravichandran-10 gene signatures) reveals promising findings in microbial differentiation in our study cohort, which includes SARS-CoV-2 patients. Nevertheless, additional validation is necessary through larger-scale studies.

The present study has some limitations: (i) A major obstacle in discovering host response biomarkers, whether single-analyte or multi-biomarker classifiers, is the lack of a universally accepted gold standard to determine the causative agent of a respiratory tract infection. (ii) For the transcriptomic biomarker analysis, a limited number of samples were used for an exploratory study, which included only the bacterial, viral, and SARS-CoV-2 categories and not samples from patients with mixed bacterial–viral etiology. However, the presence of multiple pathogens could have led to overlapping gene expression profiles, making it difficult to attribute specific changes to a particular microorganism. (iii) Another limitation is the lack of appropriate controls, for example, the inclusion of samples from patients with non-infectious causes of respiratory symptoms. This implies that our current signatures cannot be used to assist in the diagnosis of infection *per se* without further validation and rather must be interpreted in a clinical context. (iv) A small percentage of confirmed CAP cases in the viral category is also a limitation. Nevertheless, as many as 68% of the latter patients received antibiotics for more than 8 hours after admission. This highlights the challenge of accurately diagnosing the anatomical site of infection in LRTIs and underscores the need for improved methods to determine which patients require antibiotics, regardless of the infection site. (v) The cytokine/chemokine panel was pre-defined. (vi) There is a lack of external data sets to validate the performance of the identified protein signatures.

The strength of the present study lies in the meticulous collection of good-quality LRT samples and comprehensive microbiological and clinical characterization of the selected patient categories, as well as the inclusion of samples from patients with SARS-CoV-2. The results imply a promising potential for both protein and gene-based host response signatures to inform future therapeutic strategies that aim to prevent the untargeted use of antibiotics by contributing to more precise discrimination between viral and bacterial etiology in LRTIs, including CAP. Nonetheless, further development and validation in diverse clinical settings are warranted to establish the best discriminatory potential as bacterial versus viral infection host response-based classifiers, including a particular focus on the host response in COVID-19 patients.

## MATERIALS AND METHODS

### Patients and study design

This study is nested within two prospective cohorts of adult patients ≥18 years of age presenting to the Emergency Department with a suspicion of CAP. The study was conducted at Haukeland University Hospital, a tertiary care referral center in Bergen, Norway, during two time periods: 2019/2020 and 2020/2022. The first prospective cohort study (a feasibility study) enrolled 104 patients between 2 December 2019 and 17 February 2020 (pre-COVID-19 cohort) (39). The second prospective cohort enrolled 425 patients (post-COVID-19) in the context of a randomized controlled trial (NCT04660084) (40) between 25 September 2020 and 21 June 2022.

### Study procedures and sample collection

At inclusion, LRT samples (spontaneous sputum, induced sputum, or endotracheal aspirates) for microbiological analyses were obtained from all patients for pathogen detection by standard microbiological methods and the BioFire FilmArray Pneumonia panel *plus* (FAP *plus*) (bioMérieux S.A., Marcy-l’Etoile, France), as described elsewhere (41). For host biomarker analyses, peripheral blood was collected into PAXgene blood collection tubes (PreAnalytiX, Qiagen/BD Company, Hombrechtikon, Switzerland) and Vacuette blood collection tubes (Greiner bio-one International, Austria) containing EDTA to avoid coagulation. Plasma was isolated from the latter by centrifugation at 2,000 × *g* for 20 min at 4°C and stored at −80°C until the biomarker analyses.

### Clinical diagnosis

All patients presented with at least two of the following symptoms or signs: new or worsening cough, expectoration of sputum, dyspnea, hemoptysis, pleuritic chest pain, radiological evidence of pneumonia, abnormalities on chest auscultation and/or percussion, and fever (≥38.0°C). The final clinical diagnosis of RTIs was established retrospectively according to pre-defined criteria (39). A diagnosis of CAP required the presence of two or more diagnostic indicators as well as in-hospital treatment and/or a clinician-documented diagnosis of CAP, confirmed by the assessment of a study doctor. In the case of disagreement between the treating physician and the study doctor, an additional study investigator would arbitrate. Patients were classified as having an infectious exacerbation of chronic obstructive pulmonary disease (COPD) if they had no radiological evidence of pneumonia but met two diagnostic criteria and had a history of COPD exacerbation. Patients with other infections than the ones specified above, such as acute bronchitis, were merged into a third clinical category and classified as other respiratory tract infections.

### Case classification according to microbiological etiology and clinical management

We utilized results from standard microbiological methods and the BioFire FilmArray results from the analysis of LRT samples to determine the etiology of the LRTIs in individual patients. The actual clinical management defined as a decision to treat versus not treat patients with RTIs with antibiotics for a minimum of 120 hours as per national guidelines (42) was also considered.

For the identification of protein profiles, the case classifications were as follows: (i) Bacterial etiology was defined as the detection of bacteria in LRT samples, no viral detections, AND antibiotics administered for ≥120 hours (intravenously and/or oral or both, during hospitalization and/or medication prescribed on discharge). (ii) Viral etiology was defined as the microbiological detection of respiratory virus other than SARS-CoV-2, no bacterial detections, AND antibiotics administered for <120 hours in the hospital. (iii) COVID-19 was defined as the detection of SARS-CoV-2 as the only virus, no bacterial detections, AND antibiotics administered for <120 hours in the hospital. (iv) Mixed bacterial–viral etiology was defined as the microbiological detection of virus AND bacteria AND antibiotic given ≥120 hours (intravenously and/or oral or both, during hospitalization and/or medication prescribed on discharge). Patients with discordant findings [i.e., microbiological detection of only virus (including SARS-CoV-2) BUT antibiotics administered for ≥120 hours in the hospital, or microbiological detection of bacteria and/or mixed bacterial–viral (including SARS-CoV-2) in LRT samples BUT antibiotics administered for <120 hours (including post-discharge)] were excluded from this study.

For the identification of transcriptional profiles, the case classifications were defined as above; however, patients with mixed bacterial–viral etiology were not included in the transcriptional profiling analyses.

### Selected published gene signatures for bacterial–viral discrimination used for evaluation

We selected four published gene signatures from the following recent studies for evaluation: Li et al. (30) and Ravichandran et al. (29) identified 3-gene (Li-3) and 10-gene (Ravichandran-10) signatures, respectively, from cohorts that included COVID-19 patients for validation, while Xu et al. (31) and Rao et al. (32) identified 2-gene (Xu-2) and 8-gene (Rao-8) signatures that differentiated between bacterial and viral infections in cohorts where COVID-19 was not included.

### The Bio-plex assay

The Bio-plex human cytokines 27-plex panel kit (Bio-Rad Laboratories Inc., Hercules, CA, USA) was used according to the manufacturer’s instructions. The panel consists of the following biomarkers: IL1β, IL1RA, IL2, IL4, IL5, IL6, IL7, IL8/CXCL8, IL9, IL10, IL12 (p70), IL13, IL15, IL17, eotaxin/CXCL11, bFGF, granulocyte-colony stimulating factor, GM-CSF, IFNγ, IP-10/CXCL10, MCP-1/CCL2, MIP1α/CCL3, MIP1β/CCL4, platelet-derived growth factor-BB, regulated upon activation T cell expressed and secreted/CCL5, TNF, and VEGF. Data acquisition was performed on a Luminex100 analyzer (Luminex Corporation, Austin, Texas, USA) according to the manufacturer’s instructions. Cytokine/chemokine concentrations were measured in pg mL^−1^.

### Standard laboratory tests

CRP and PCT were measured in serum samples within 48 hours of hospital admission using immunoturbidimetric and electrochemiluminescence immunoassay methods, respectively. The results from these protein biomarkers obtained by routine analyses were combined with data from the bio-plex assay in analyses of discriminatory host protein profiles between the clinical groups.

### Total RNA extraction

Total RNA concentration and purity were measured using a Nanodrop spectrophotometer (Thermo Scientific, Wilmington, DE, USA) and ranged between 2.2 and 15.5 µg (average 6.7 ± 3.85 µg). In addition, RNA integrity number was evaluated using an Agilent 2100 Bioanalyzer.

### Clariom S assay

Clariom S Human Assay (Thermo Fisher Scientific) was performed according to the manufacturer’s instructions. Sample labeling and hybridization were undertaken according to the GeneChip WT PLUS Reagent Kit User Manual (Thermo Fisher Scientific). The array was scanned with the GeneChip Scanner 3000 7G (Thermo Fisher Scientific) by the GeneChip Command Console AGCC 4.0 User Manual (Thermo Fisher Scientific). The transcriptomic data are publicly available from NCBI GEO (www.ncbi.nlm.nih.gov/geo/) under super-series accession GSE236318.

Gene expression profiles were measured using Transcriptome Analysis Console (TAC) software (version 4.0.2; Applied Biosystems, Foster City, CA, USA). The significantly differentially expressed genes between each pairwise comparison were extracted based on the following default criteria: fold change, ≥1.0- or ≤−1.0-fold; *P* < 0.05 {one-way analysis of variance; exact *P*-values [obtained using an exact test using R package edgeR (43) within the TAC software]}. In addition, PCA plots, volcano plots, hierarchical clustering, and the distribution of the top 30 gene sets were generated using TAC software. Metascape is a powerful tool for gene function annotation analysis (44) used to analyze batch genes and proteins to understand the cognition of gene or protein functions. It combines numerous reliable functional databases, including GO, Kyoto Encyclopedia of Genes and Genomes, Wiki pathways, and Uniprot, to analyze data sets with multiple genes, and uses Cytoscape to visualize enrichment networks.

### Statistical analysis

Patient characteristics were summarized using the mean and minimum/maximum or count and percentage, as appropriate. Pairwise comparisons (Kruskal–Wallis with Dunn’s correction test) were performed between the clinical groups of different etiologies: bacterial (*n* = 36), viral (*n* = 27), SARS-CoV-2 (*n* = 22), and mixed bacterial–viral etiology (*n* = 38). The Lasso regression model was applied to identify the best discriminatory biosignature. As described in other studies (45, 46), optimal tuning parameters were found using a cross-validation step repeated 100 times to stabilize the results. A predicted probability of <0.5 resulted in the classification of bacterial etiology, whereas >0.5 resulted in the classification of viral etiology. This model-based classification was compared to participants’ actual “true” classification, and the number of correctly classified participants could be identified. Specifically, the predictive abilities of the signatures (to classify participants correctly) in both training and test sets were summarized utilizing receiver operator characteristic (ROC) curves, sensitivity, specificity, and AUC. Analyses were conducted using glmnet, pROC packages in R (R Core Team) through the graphical user interface RStudio (www.rstudio.com) (47).

A logistic regression was conducted utilizing gene signatures from studies of other research groups (29–32) to discriminate between bacterial etiology and viral + SARS-CoV-2 etiology in our study samples. To validate the precision of the results, a ROC curve was constructed using SPSS 28.

## SUPPLEMENTAL MATERIAL

Figure S1 spectrum.02831-23-s0001.tiff ROC curves for protein signature.

Figure S2 spectrum.02831-23-s0002.tiff ROC curves for the evaluation of protein signature.

Table S1 spectrum.02831-23-s0003.xlsx Differentially expressed genes in patients with bacterial etiology.

ASM does not own the copyrights to Supplemental Material that may be linked to, or accessed through, an article. The authors have granted ASM a non-exclusive, world-wide license to publish the Supplemental Material files. Please contact the corresponding author directly for reuse.
