# Supplementary figures and images for: Protein and transcriptional biomarker profiling may inform treatment strategies in lower respiratory tract infections by indicating bacterial–viral differentiation
Source: Microbiol Spectr. 2024 Sep 13;12(10):e02831-23. doi: 10.1128/spectrum.02831-23 (PMC11448388; doi:10.1128/spectrum.02831-23)

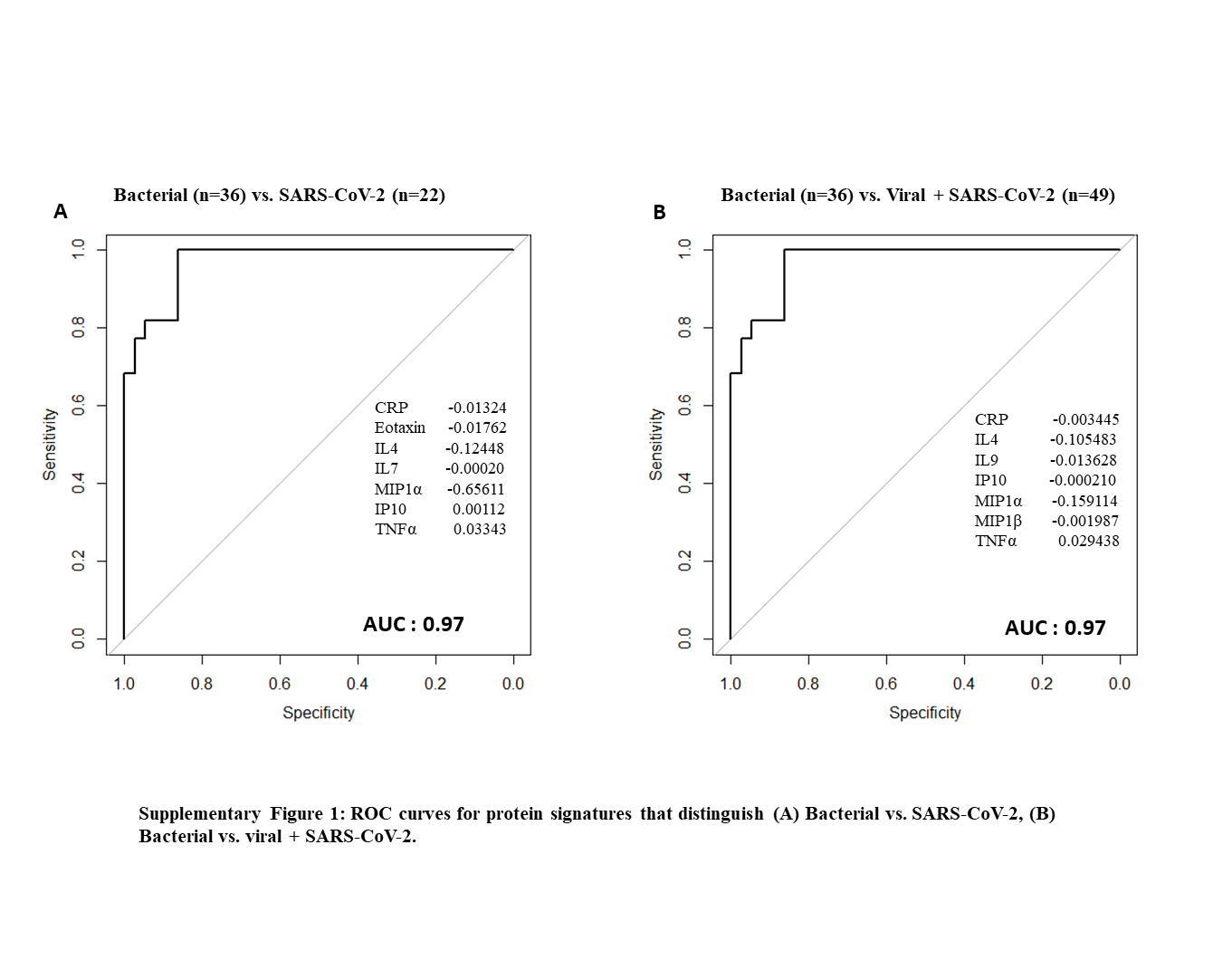

Supplement: Figure S1 — ROC curves for protein signature. [file spectrum.02831-23-s0001.tiff]

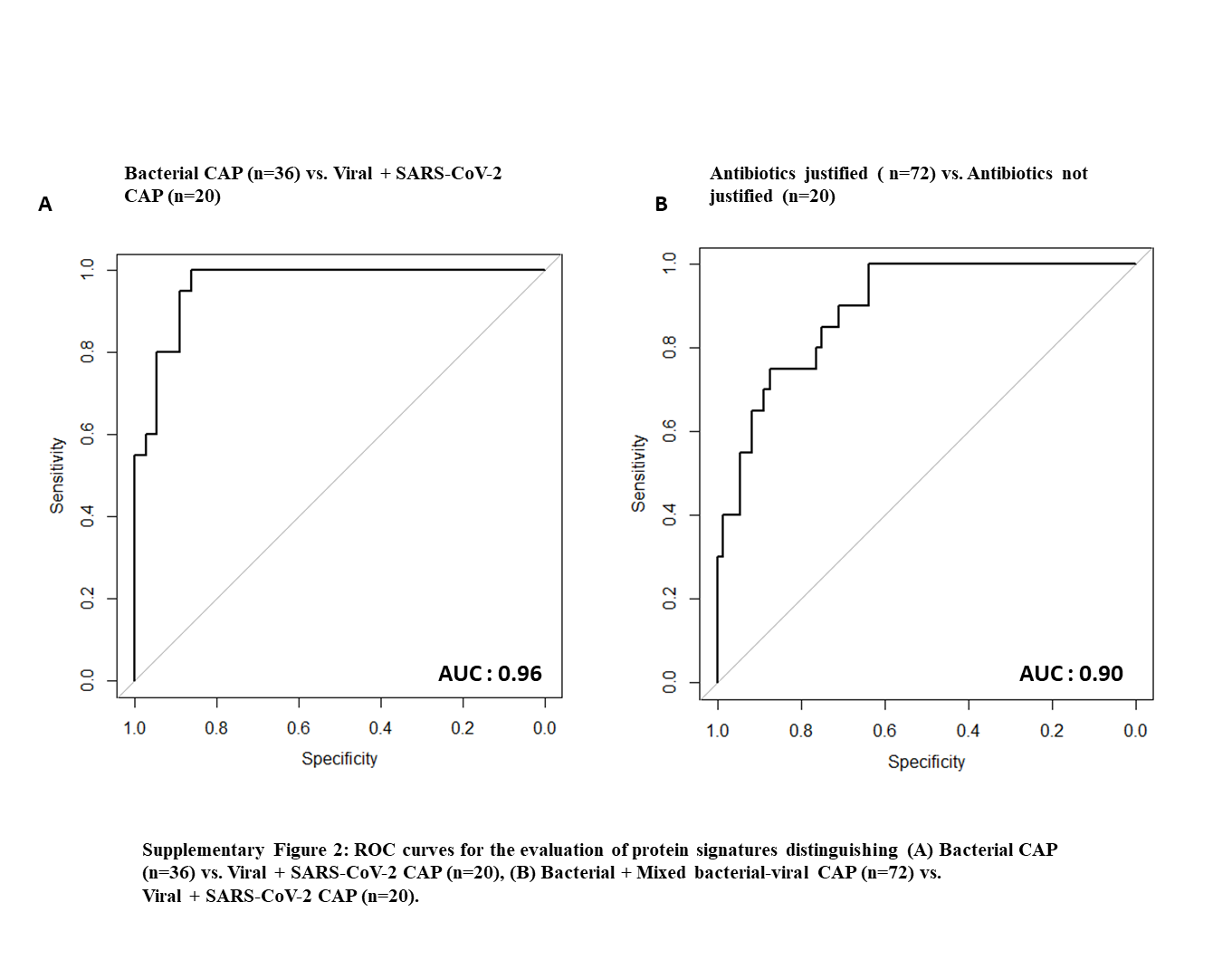

Supplement: Figure S2 — ROC curves for the evaluation of protein signature. [file spectrum.02831-23-s0002.tiff]
